# Seromucinous Hamartoma of the Nasal Cavity: A Rare Entity Presenting a Diagnostic Challenge in Preoperative Biopsy

**DOI:** 10.3390/diagnostics16101452

**Published:** 2026-05-10

**Authors:** Taimei Egashira, Masayoshi Kobayashi, Eisuke Ishigami

**Affiliations:** Department of Otorhinolaryngology-Head and Neck Surgery, Mie University Graduate School of Medicine, 2-174 Edobashi, Tsu 514-8507, Mie, Japan; m-koba@doc.medic.mie-u.ac.jp (M.K.); ishigami0610@med.mie-u.ac.jp (E.I.)

**Keywords:** biopsy diagnosis, diagnostic challenge, endoscopic endonasal surgery, seromucinous hamartoma, nasal cavity

## Abstract

**Background/Objectives**: Seromucinous hamartoma (SH) is an extremely rare benign glandular lesion arising in the nasal cavity and paranasal sinuses, characterized by proliferation of serous and mucinous glands. Preoperative diagnosis by biopsy is extremely uncommon, making it a diagnostic challenge. We report a case of SH and discuss its diagnostic difficulties and management. **Case Presentation**: A 52-year-old man presented with right-sided nasal obstruction. A lobulated mass in the posterior right nasal cavity was incidentally detected during transnasal endoscopy. The lesion persisted for one year without reduction. CT, MRI, and biopsy failed to provide a definitive diagnosis. The patient was referred to our department, and endoscopic surgery under general anesthesia was performed. The tumor was removed en bloc. Histopathological examination revealed glandular proliferation of mixed serous and mucinous glands within the subepithelial stroma, consistent with SH. **Discussion**: Preoperative diagnosis is difficult due to insufficient biopsy depth and limited recognition of this rare entity. Since the surface epithelium shows no atypia, identification of subepithelial glandular proliferation is essential. Larger and deeper biopsy specimens and communication with pathologists may improve diagnostic yield. Surgical excision is the treatment of choice. As SH often arises in the posterior nasal cavity and is highly vascular, en bloc resection under general anesthesia is recommended. **Conclusions**: Recognition of SH is important to improve diagnostic accuracy. Appropriate biopsy strategy and surgical planning based on tumor location are essential.

## 1. Introduction

Hamartomas are benign, tumor-like malformations characterized by a disorganized proliferation of mature tissue elements that are native to the anatomical site in which they arise. They are generally distinguished from true neoplasms by the absence of destructive infiltrative growth and by their composition of differentiated, non-atypical cells. In the sinonasal tract, hamartomatous lesions are uncommon, and they encompass several clinicopathological entities, including respiratory epithelial adenomatoid hamartoma (REAH), chondro-osseous respiratory epithelial adenomatoid hamartoma (COREAH), and seromucinous hamartoma (SH) [1,2,3]. Among these, SH is one of the rarest and remains unfamiliar to many clinicians and pathologists.

SH is characterized histologically by a proliferation of small serous and mucinous glands in the subepithelial stroma beneath an intact, non-atypical respiratory epithelium [4,5]. Since its recognition as a distinct lesion, only a limited number of cases have been reported worldwide. Even after formal inclusion in the 4th Edition of the WHO Classification of Head and Neck Tumours in 2017 [6] and continued recognition in the 5th Edition in 2024 [7], the cumulative number of documented cases has remained low. This persistent scarcity is important not only because it underscores the rarity of the lesion itself, but also because it suggests that SH may be underrecognized in routine clinical practice and underdiagnosed in small biopsy specimens.

From a clinical perspective, SH presents a diagnostic dilemma. Most reported cases have arisen in the posterior nasal cavity, choanal region, or nasopharynx and have manifested as nonspecific symptoms such as nasal obstruction, rhinorrhea, epistaxis, or postnasal discomfort [8,9]. In some patients, the lesion has been discovered incidentally during endoscopy performed for unrelated reasons. Radiological findings are similarly nonspecific. Computed tomography usually shows a well-circumscribed soft-tissue lesion without aggressive bony changes, while magnetic resonance imaging may demonstrate signal characteristics similar to surrounding mucosa [10]. Because neither symptoms nor imaging findings are pathognomonic, histopathological evaluation is essential for diagnosis. However, paradoxically, preoperative diagnosis is rarely achieved.

This discrepancy between the theoretical centrality of biopsy and its limited practical utility is one of the most important clinical issues surrounding SH. In many reported cases, a biopsy either suggested a nonspecific inflammatory lesion, a hamartomatous polyp, or a benign glandular process without permitting a definitive diagnosis [8,11,12,13,14,15,16,17,18,19,20,21,22]. The reasons for this diagnostic limitation have not been sufficiently analyzed in detail, although they are highly relevant to daily clinical practice. A better understanding of why biopsy fails is valuable because it may help clinicians optimize tissue sampling, improve communication with pathologists, and select a more appropriate operative strategy.

Another important aspect of SH is its histopathological overlap with other sinonasal glandular lesions. The differential diagnosis is broad and includes not only inflammatory polyps associated with chronic rhinosinusitis, sinonasal papilloma, angiofibroma, and fibrous lesions, but also REAH, low-grade sinonasal adenocarcinoma, and, in some cases, seromucinous carcinoma or other low-grade glandular neoplasms [5,12,13,14,15,23]. These lesions differ greatly in biological behavior and therapeutic implications. Therefore, even though SH itself is benign, accurate diagnosis is essential in order to avoid both undertreatment of malignant disease and overtreatment of a benign lesion.

In the present report, we describe a case of SH arising in the posterior nasal cavity that could not be diagnosed preoperatively despite biopsy. We focus not only on the clinical, radiological, and histopathological findings of the case itself, but also on the clinicopathological reasons that made diagnosis difficult. In addition, we review the relevant literature to clarify how this case contributes to current knowledge. Specifically, we argue that the preoperative diagnostic difficulty of SH is closely related to two factors: the limited depth and representativeness of routine biopsy specimens and the insufficient recognition of this entity among both clinicians and pathologists. By highlighting these points, we aim to provide practical guidance for improving diagnostic accuracy in future cases.

Additionally, surgical treatment was performed in the present case. With regard to the surgical approach, endoscopic surgery has been selected in most reported cases because SH is a benign tumor, tends to arise in the deep nasal cavity, and often lacks a definitive preoperative diagnosis. Through the present case, we also discuss and review the available surgical treatment options for this lesion.

## 2. Case Presentation

A 52-year-old man was referred to our department with a complaint of persistent right-sided nasal obstruction. One year before referral, a lesion in the posterior part of the right nasal cavity had been noted incidentally during an upper gastrointestinal endoscopy performed through the nasal route as part of a routine health checkup. At that time, he had no severe sinonasal symptoms and did not undergo definitive treatment. He subsequently visited a local otorhinolaryngology clinic, where endoscopic examination demonstrated a smooth, pedunculated mass in the posterior right nasal cavity. Because the lesion did not decrease in size during approximately six months of observation, he was referred to a community hospital for further evaluation. At the community hospital, computed tomography, magnetic resonance imaging, and tissue biopsy were performed. Although the lesion was considered to be benign-appearing on imaging, the biopsy did not yield a definitive diagnosis. The patient continued to experience right-sided nasal obstruction and was therefore referred to our institution for further evaluation and management. The patient had comorbid allergic rhinitis. Serum allergen-specific IgE antibody testing performed at our institution showed elevated levels for *Dermatophagoides pteronyssinus* (2.16 UA/mL), house dust (1.84 UA/mL), Japanese cedar (12.00 UA/mL), and cypress (0.83 UA/mL) (only elevated values are shown). There was no history of intranasal medication use. His past medical history included cholecystectomy for acute cholecystitis and appendectomy for acute appendicitis. There was no history of prior sinonasal surgery or trauma, chronic rhinosinusitis requiring surgery, autoimmune disease, or head and neck malignancy. On initial examination in our department, laboratory findings showed a serum squamous cell carcinoma antigen level of 0.8 ng/mL, total IgG of 1652 mg/dL, and serum IgG4 of 104 mg/dL. These findings did not suggest an epithelial malignancy or IgG4-related disease. Other routine blood tests were unremarkable.

**Figure 1:**
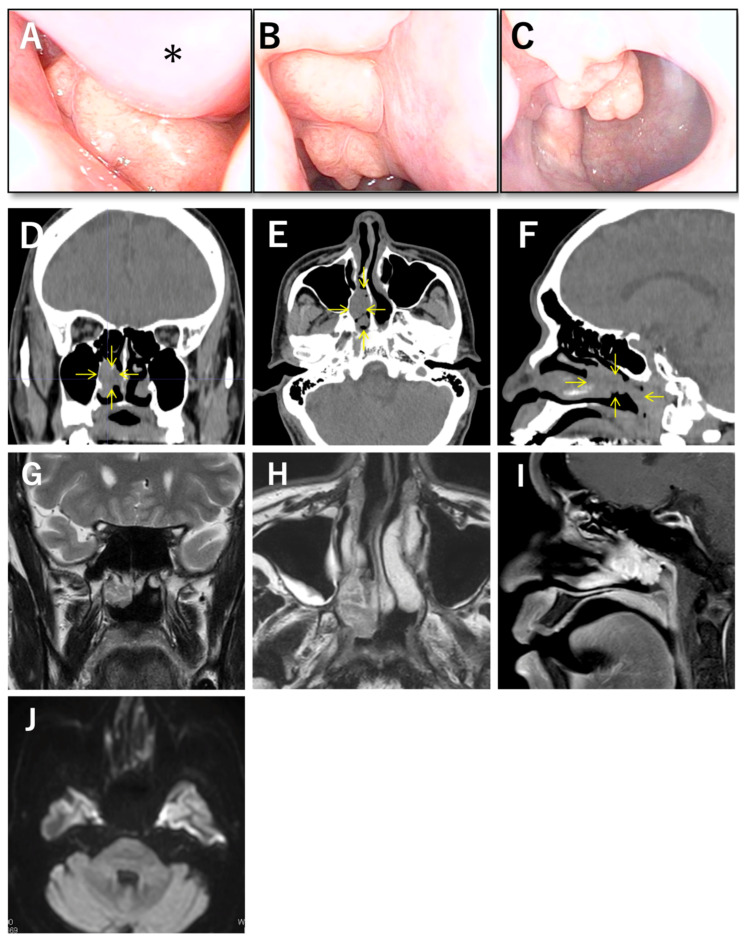
(**A**) Endoscopic view showing the anterior surface of the right nasal cavity mass. The asterisk (*) indicates the right middle turbinate. (**B**) The mass appears milky-white, smooth-surfaced, and lobulated. (**C**) The posterior end of the mass extends to the choana. (**D**–**F**) Coronal, axial, and sagittal non-contrast CT images of the paranasal sinuses showing a soft-tissue-density lesion measuring approximately 28 mm in the posterior right nasal cavity, without evidence of adjacent bone destruction or localized sclerosis. (**G**,**H**) Coronal, axial, and sagittal MRI findings. On T2-weighted images, the lesion showed a heterogeneous low-signal-intensity area. (**I**) On contrast-enhanced T1-weighted images, faint enhancement was observed mainly along the lobulated surface of the mass; however, there was no marked difference compared with the surrounding nasal mucosa of the septum and middle turbinate. (**J**) Diffusion-weighted imaging revealed no restricted diffusion within the lesion.

Nasal endoscopy demonstrated rightward septal deviation with narrowing of the right nasal cavity. Beyond the narrowed space, a smooth-surfaced, milky-white, lobulated mass was visible in the posterior right nasal cavity (Figure 1A–C). The lesion appeared pedunculated and extended posteriorly toward the choana. The surface was intact, without ulceration, apparent necrosis, or friability. There was no obvious purulent discharge, and the surrounding mucosa showed no severe inflammatory change. Non-contrast computed tomography demonstrated a soft-tissue-density lesion measuring approximately 28 mm in the posterior right nasal cavity (Figure 1D–F). The lesion was relatively well circumscribed. Importantly, there was no evidence of bone destruction, adjacent hyperostosis, or invasive change into neighboring structures. Septal deviation with spur formation to the right side was also observed. These findings were consistent with a localized lesion but did not permit specific characterization. Magnetic resonance imaging was subsequently reviewed in detail. The lesion appeared lobulated. On T1-weighted images, it showed relatively high signal intensity compared with surrounding soft tissue. On T2-weighted images, it showed heterogeneous low signal intensity (Figure 1G,H). On contrast-enhanced T1-weighted images, faint enhancement was observed mainly along the lobulated surface of the mass, but there was no marked difference from the enhancement pattern of the normal nasal mucosa, including the middle turbinate and nasal septum (Figure 1I). Diffusion-weighted imaging showed no restricted diffusion, and the apparent diffusion coefficient was preserved (Figure 1J). Collectively, the imaging findings suggested a nonaggressive lesion, but they were not sufficiently specific to establish a definitive diagnosis. Differential diagnostic considerations at this stage included a benign polypoid lesion, a hamartomatous lesion, or a low-grade glandular lesion.

Histopathological examination of the biopsy specimen obtained at the referring hospital demonstrated ciliated respiratory epithelium and edematous connective tissue with mild infiltration of lymphocytes and plasma cells. However, no definite neoplastic component or characteristic glandular proliferation was identified. Thus, the biopsy was regarded as nondiagnostic. Because the patient desired definitive treatment for nasal obstruction and because a precise pathological diagnosis remained uncertain, complete surgical excision was planned.

Under general anesthesia, endoscopic endonasal surgery was performed. To improve the surgical field, septoplasty was first carried out via a Killian incision in the left nasal cavity. After correction of the septal deviation, the posterior right nasal cavity could be visualized more clearly. A pedunculated tumor occupying the posterior right nasal cavity was identified. Intraoperatively, the tumor was found to originate from the posterior aspect of the middle meatus. It extended laterally and posteriorly along the inferior surface of the middle turbinate and reached the area superior to the pharyngeal orifice of the eustachian tube. The stalk was relatively broad, but no adhesion to the nasal septum or nasopharyngeal mucosa was present.

**Figure 2:**
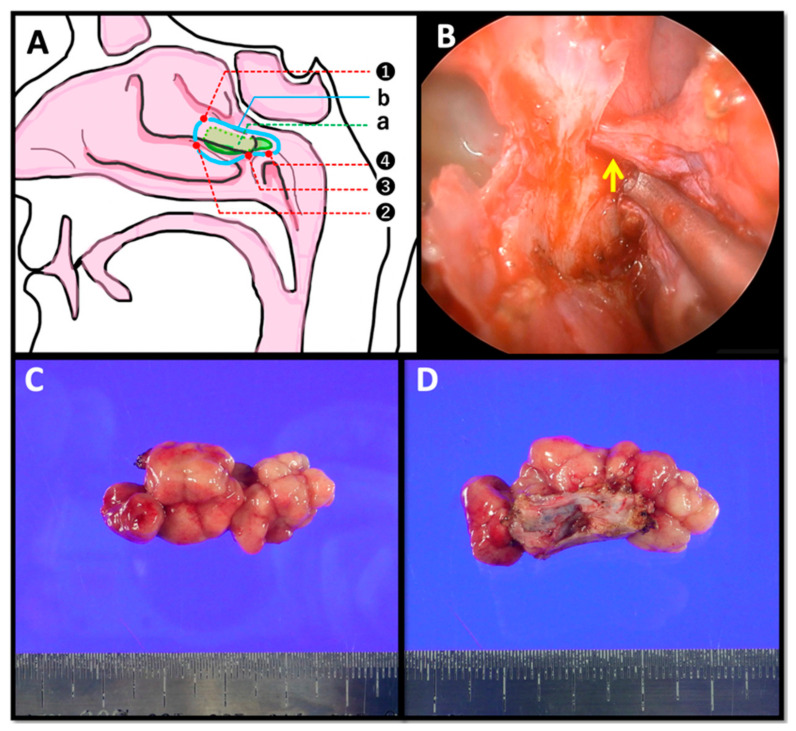
(**A**) Intraoperative view illustrating the tumor base and biopsy sites. Region **a** corresponds to the area of tumor attachment. Biopsy specimens were obtained from four sites: (1) the anterolateral portion of the nasal lateral wall adjacent to the tumor, (2) the anteroinferior portion of the nasal lateral wall adjacent to the tumor, (3) the posteroinferior end of the middle turbinate, and (4) the superior aspect of the torus tubarius. The mucosal resection line connecting these four points is indicated by **b**. (**B**) Intraoperative view during submucosal dissection. The sphenopalatine artery crossing the resection line was identified and transected. (**C**,**D**) Gross findings of the resected tumor. (**C**) The tumor was removed en bloc, measuring 35 × 18 × 15 mm. The lateral aspect shows the surface attached to the lateral nasal wall. (**D**) Opposite view of (**C**), showing a lobulated tumor without adhesion to the surrounding mucosa.

Because malignancy had not been definitively excluded preoperatively, the extent of mucosal resection was determined carefully. Mucosal samples were obtained from four sites surrounding the tumor base for intraoperative frozen-section analysis: the anterolateral portion of the nasal lateral wall adjacent to the tumor, the anteroinferior portion of the nasal lateral wall adjacent to the tumor, the posteroinferior end of the middle turbinate, and the superior aspect of the torus tubarius. None of these specimens showed evidence of malignancy on frozen section (Figure 2A). Based on these findings, the lesion was resected endoscopically in an en bloc fashion. The mucosa was dissected from the underlying bone, and the posterior-inferior portion of the middle turbinate was removed together with the tumor. During the dissection, a cord-like structure containing the sphenopalatine artery crossed the planned resection line (Figure 2B). This vessel was identified clearly and transected in a controlled manner. Complete hemostasis was achieved, and the lesion was removed safely without major complication. Grossly, the resected specimen measured 35 × 18 × 15 mm and had a lobulated appearance (Figure 2C,D).

**Figure 3:**
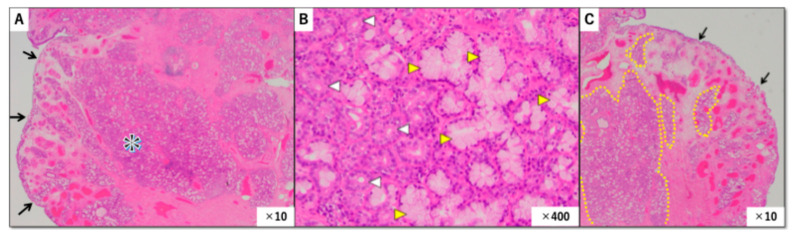
(**A**,**B**) Histopathological features of the lesion (hematoxylin and eosin staining). (**A**) Low-power view showing edematous tissue covered by ciliated columnar epithelium (arrows). The subepithelial stroma exhibits glandular proliferation composed of mixed eosinophilic and goblet cells forming densely basophilic areas. The region marked with an asterisk (*) is shown at higher magnification in (**B**). (**B**) High-power view of the area indicated by an asterisk in (**A**), showing irregular proliferation of both mucous glands (yellow arrowheads ▷) and serous glands (white arrowheads ◁). (**C**) Low-power view from another area of the same lesion. The surface epithelium (arrows) shows no atypia. For accurate histopathological evaluation, biopsy specimen should include the subepithelial glandular proliferative area outlined by the dotted line.

Postoperative histopathological examination of the resected specimen revealed edematous tissue covered by ciliated columnar epithelium. Within the subepithelial stroma, there was irregular proliferation of numerous small glandular structures composed of mixed serous and mucinous elements (Figure 3A). The glandular cells showed no cytologic atypia, no mitotic activity, and no invasive growth pattern (Figure 3B). These findings were diagnostic of seromucinous hamartoma.

The postoperative course was uneventful. The patient’s nasal obstruction improved, and follow-up endoscopy demonstrated satisfactory healing of the surgical site. No evidence of recurrence was observed at six months after surgery.

## 3. Discussion

Seromucinous hamartoma is a rare benign lesion of the sinonasal tract, and, because of its rarity, each well-documented case can contribute meaningfully to the literature. The present case is notable not merely because SH itself is uncommon, but because it illustrates in a concrete manner why preoperative diagnosis is so difficult and how clinicians may improve the diagnostic process.

### 3.1. Historical and Pathological Context

The lesion now known as SH was originally described several decades ago [4], but recognition of the entity has developed slowly. This slow accumulation of knowledge is reflected in the relatively small number of reported cases even after formal inclusion in the WHO classification. The persistent rarity may indicate genuine low incidence, but it may also reflect underrecognition, inconsistent nomenclature in older publications, and diagnostic confusion with related lesions.

Histologically, SH consists of a proliferation of glands showing serous and mucinous differentiation within the subepithelial stroma beneath a non-atypical respiratory epithelial surface [7]. The lesion is benign, and its architectural appearance is usually much more important diagnostically than any single cytological feature. In contrast to malignant glandular lesions [12,13,14,15], SH lacks cytological atypia, destructive invasion, and complex infiltrative architecture. In contrast to some other hamartomatous lesions, its hallmark is the glandular composition itself.

### 3.2. Why Preoperative Biopsy So Often Fails

The central message of this case is that routine biopsy may fail because the most diagnostic component of SH lies deeper than the tissue commonly obtained in superficial endoscopic sampling. In our case, the biopsy specimen demonstrated only respiratory epithelium and edematous stroma with mild inflammatory infiltrate. These are nonspecific findings that could be seen in many benign sinonasal conditions. The resected specimen, by contrast, clearly demonstrated the subepithelial proliferation of mixed serous and mucinous glands required for diagnosis (Figure 3C).

This discrepancy is not incidental; it reflects the histological anatomy of the lesion. SH is usually covered by relatively bland respiratory mucosa. Therefore, a forceps biopsy that samples only the surface or immediate subepithelial layer may miss the representative glandular proliferation entirely. This point deserves emphasis because, in daily practice, many benign-appearing posterior nasal lesions are biopsied conservatively, especially when access is limited and the operator wishes to avoid bleeding. Such an approach is understandable, but it may lead directly to nondiagnostic pathology in SH.

The technical difficulty is compounded by lesion location. Many SHs arise in the posterior nasal cavity or adjacent regions [8,9], where the working angle may be narrow and where adjacent vascular structures are present. Under those conditions, a clinician may understandably prefer a limited sample. However, the present case suggests that if SH is considered in the differential diagnosis, efforts should be made to obtain a larger and deeper specimen, ideally including sufficient subepithelial tissue. In practical terms, this may mean using a more substantial instrument, targeting the lesion base, or considering biopsy under more controlled conditions when office-based sampling is unlikely to be representative.

### 3.3. The Importance of Disease Recognition

The second major factor in diagnostic difficulty is limited awareness of SH. Rare diseases are, by definition, less likely to be considered in routine differential diagnosis. This affects both the clinician who selects the biopsy site and the pathologist who interprets the specimen. If SH is not suspected clinically, the biopsy may be too superficial. If SH is not considered histologically, even a partially representative specimen may be interpreted as nonspecific glandular or inflammatory change. This is why communication between surgeon and pathologist is particularly important in rare sinonasal lesions. When the clinician communicates that a posterior nasal lesion is smooth, lobulated, benign-appearing, and difficult to classify radiologically, the pathologist may be more likely to actively consider entities such as SH or REAH. Similarly, if the pathologist recognizes that the biopsy is superficial and nondiagnostic rather than definitively benign in a specific way, the surgical team may be more willing to pursue a definitive excisional strategy.

In our case, retrospective comparison of the biopsy and resection specimens strongly suggests that awareness alone would not have been sufficient without adequate tissue, but adequate tissue alone also might not have guaranteed diagnosis unless the lesion was actively considered. Thus, tissue adequacy and disease recognition should be viewed as complementary rather than competing explanations.

### 3.4. Review of Reported Clinical Characteristics

The literature suggests several broad clinical tendencies in SH. Reported patients have included adults across a range of ages, with no universally accepted sex predilection because the total number of cases is small [8,21]. Symptoms are usually nonspecific and depend largely on lesion size and location. Nasal obstruction appears to be one of the most common complaints. Some lesions are discovered incidentally, which is not surprising given their benign nature and often slow growth.

The posterior nasal cavity and neighboring regions are common sites of origin [8,9,21]. This distribution is clinically meaningful because lesions in these areas are more difficult to assess thoroughly in the office and may be more difficult to biopsy representatively. The posterior location also influences surgical strategy: a lesion that appears small on imaging may still require general anesthesia and endoscopic resection for safe and complete management.

Radiologically, the available reports do not support a single pathognomonic pattern. Nevertheless, SH often appears as a localized mass without aggressive features such as bone destruction, marked infiltrative margins, or restricted diffusion. In the present case, the preservation of the apparent diffusion coefficient and the absence of destructive CT findings were reassuring and consistent with a benign process. Still, imaging alone could not distinguish SH from other benign or low-grade lesions.

### 3.5. Differential Diagnosis

The differential diagnosis of SH is broad enough to be clinically important. Among benign lesions, REAH is probably the most relevant comparator. REAH is also a glandular lesion of the sinonasal tract and can present as a polypoid mass. Histologically, however, REAH is characterized by glandular invaginations lined by ciliated respiratory epithelium and often accompanied by thickened, hyalinized basement membranes [5]. SH, in contrast, shows proliferation of smaller seromucinous glands in the stroma without the same type of prominent thickened basement membrane. The distinction can usually be made on routine hematoxylin-eosin sections when the specimen is adequate, but in small biopsies the two may be confused or incompletely represented.

A second important differential consideration is low-grade sinonasal adenocarcinoma or related low-grade glandular neoplasia. These malignant lesions may also show gland formation and may appear deceptively bland in some areas. However, they generally exhibit more complex architecture, more evident cytological abnormality, and some degree of destructive or infiltrative growth [12,13,14,15]. SH lacks these malignant features. In practice, when the pathology is equivocal and the biopsy is limited, the absence of definitive malignancy does not necessarily establish a benign diagnosis; instead, it may simply indicate inadequate sampling.

Malignant transformation to low-grade adenocarcinoma, although rare, is also worth mentioning. Its relationship to benign seromucinous proliferations remains a topic of interest, and one report of malignant transformation arising in association with SH has heightened awareness of this possibility [24]. Although such transformation appears exceedingly rare, the existence of that report supports complete excision and careful histological assessment when a lesion remains unclassified preoperatively.

Immunohistochemistry may be useful in selected difficult cases. Reports have described the utility of markers such as p63 and S100 in distinguishing SH from other lesions, particularly when REAH is a consideration [5,23]. However, in a typical resection specimen, routine morphology is often sufficient. Immunohistochemistry should therefore be regarded as an adjunct rather than a substitute for adequate sampling and careful histological interpretation.

### 3.6. Surgical Management and Rationale for Complete Excision

Because preoperative diagnosis is often inconclusive, surgical excision frequently serves both diagnostic and therapeutic purposes. This dual role is especially important in SH. In a patient with persistent symptoms, a benign-appearing but unclassified lesion, and nondiagnostic biopsy, complete excision is a rational strategy. It provides definitive tissue diagnosis, relieves symptoms, and removes any residual uncertainty about a possible low-grade neoplasm.

The present case also highlights an operative point that deserves mention in expanded discussion: posterior nasal lesions may be associated with meaningful vascularity. In our patient, a branch of the sphenopalatine artery crossing the resection field had to be managed intraoperatively. This supports the recommendation that lesions suspected to be SH, especially when posteriorly located, are often better managed under general anesthesia in a controlled endoscopic setting rather than through limited outpatient removal. En bloc excision is preferable because it preserves lesion architecture, maximizes diagnostic accuracy, and reduces the risk of leaving behind residual tissue.

Regarding the surgical procedure, rigid endoscopy provided sufficient access to the tumor and allowed the operation to be performed without difficulty in maneuverability. The factor that determines the limitation of the endoscopic approach is not the accessibility itself, but rather the pathological nature of the tumor. If the lesion is malignant, not only the tumor itself but also the surrounding structures must be resected with an adequate pathological safety margin. In such cases, the feasibility of achieving en bloc resection using an endoscopic approach alone defines the practical limitation of endoscopic surgery.

To ensure an adequate surgical margin for en bloc resection without leaving residual tumor tissue, it is safer to harvest mucosa surrounding the tumor base and perform intraoperative frozen-section pathological examination. For this purpose, it is essential to first carefully assess the entire extent of tumor occupancy, determine whether there is adhesion to surrounding structures, and identify the precise range of the tumor base. Adequate visualization and working space are therefore indispensable. In the present case, septoplasty was performed first to secure an adequate surgical field, followed by detailed endoscopic observation of the tumor.

In this case, mucosa surrounding the tumor base was initially sampled, after which the resection line was established by connecting areas confirmed to be tumor-negative, allowing subperiosteal mucosal dissection on the inner side of the planned margin. However, in larger tumors or in cases where the tumor base is obscured behind the tumor itself and difficult to visualize, it may not be possible to establish a continuous resection line from the outset. In such situations, surgical manipulation should proceed gradually from the anterior area where visualization can be secured, while confirming adequate safety margins using intraoperative frozen-section pathology. As the surgical field and working space improve, additional frozen-section specimens can be obtained from deeper mucosa, and the resection line can then be extended and connected sequentially, facilitating safer en bloc tumor removal.

Postoperative outcomes are generally favorable. SH is benign, and recurrence appears to be uncommon after complete excision [23]. In our patient, the postoperative course was uncomplicated and no recurrence was seen during follow-up. Although longer surveillance may be prudent, the prognosis after complete removal appears excellent.

### 3.7. Contribution of This Case and Practical Recommendations

The significance of the present case lies in clearly demonstrating the discrepancy between a nondiagnostic superficial biopsy and a diagnostic resection specimen, and in explaining this discrepancy by the characteristic subepithelial location of the glandular proliferation that defines seromucinous hamartoma (SH). In addition, this case highlights that rarity itself constitutes a diagnostic hazard: SH may be overlooked not because it is inherently difficult to recognize, but because it is not actively considered. Taken together, these findings suggest that SH should be included in the differential diagnosis of smooth, lobulated, benign-appearing posterior nasal lesions, particularly when imaging indicates a localized nonaggressive process. Clinicians should be aware of the limitations of superficial biopsy and make efforts to obtain adequately deep tissue samples that include subepithelial stroma. Close communication between clinicians and pathologists is also essential to improve diagnostic accuracy. When biopsy remains inconclusive and the lesion is symptomatic or persistent, complete endoscopic excision under general anesthesia is both a reasonable and often preferable strategy for diagnosis and treatment. Histopathological evaluation should be based on a comprehensive assessment of glandular distribution and type, stromal context, cytological blandness, and the absence of infiltrative growth.

## 4. Conclusions

Seromucinous hamartoma is a rare benign glandular lesion of the sinonasal tract that poses a disproportionately large diagnostic challenge. The present case demonstrates that preoperative biopsy may fail because the defining pathological features lie within the subepithelial stroma and may be missed by superficial sampling. It also shows that limited disease recognition among clinicians and pathologists contributes substantially to diagnostic difficulty. Greater awareness of SH, combined with deliberate efforts to obtain adequate tissue and close communication between surgeon and pathologist, may improve preoperative diagnosis. When diagnostic uncertainty persists, complete endoscopic excision is both an effective treatment and the most reliable means of achieving definitive diagnosis.

## Data Availability

Data supporting the findings of this study are available from the corresponding author upon reasonable request.

## Abbreviations

The following abbreviations are used in this manuscript:

SH	Seromucinous hamartoma
REAH	Respiratory epithelial adenomatoid hamartoma
COREAH	Chondro-osseous respiratory epithelial adenomatoid hamartoma
MRI	Magnetic resonance imaging
CT	Computed tomography
WHO	World Health Organization
